# The Pious Physician, or the Claims of Religion upon the Medical Profession

**Published:** 1859-03

**Authors:** B. M. Palmer

**Affiliations:** Pastor


*The Pious Physician, or the Claims of Religion upon the Medical Profession*.—A Discourse delivered upon the occasion of the death of the late JOHN M. W. PICTON, M. D., in the First Presbyterian Church, New Orleans, Dec. 26th, 1858, by B. M. PALMER, D. D., Pastor.

TEXT—"Brethren, be followers together of me, and mark them which walk so, as ye have us for an example."—*Philippians, 3d ch., 17th v.*

Through ten successive Sabbaths this drapery of mourning has been presented to your view, a fit emblem of the deeper sorrow which has shrouded our hearts. This earth can sustain no greater loss than in the death of a truly good man. There is one example less of successful virtue encountering the temptations of life; one illustration less of the power of principle nerving to the discharge of duty; one instance less of heroic self-denial in living for the good of others; and one exhibition less of that passive fortitude which endures bereavement and misfortune. The extinction of a single light must be deplored in a world so darkened by ignorance and sin as ours; and tears of honest grief must fall upon the graves of those whose lives were a continued exposition of the precepts of virtue and the maxims of religion. All this, in all its breadth, may be said of our deceased brother, in memory of whom this Church is now dressed in the weeds of mourning.

Dr. John M. W. Picton was born in the town of Woodberry, N. J., on the 19th November 1804. Blessed with pious parents, the seeds of religious knowledge were early sown, which, though long dormant, bore abundant fruit in his latter years. His character was moulded under the influence of a mother, who was eminent alike for her intellectual attainments, and for her deep piety. His father, a Clergyman in the Presbyterian Church still survives in the enjoyment of a green old age, bowing down under this heavy sorrow, and exclaiming with the pathos of David, "My son, my son, would to God I had died for thee."

At the early age of fourteen he was entered a pupil in the military school at West Point; when, notwithstanding his youthfulness, he sustained himself with credit, graduating with distinction, possessing alike the love of his associates, and the esteem of his Preceptors. Having requited the State by seven years' service in the army, he was married in his twenty-sixth year, and selected the medical profession as his future calling. Two years of laborious study in the Medical school at Philadelphia prepared him to enter upon the practice of physic in this city; where he soon distinguished himself, attracting the attention of the entire faculty, not only in this country, but also in Europe, by the splendid success of some surgical operations. Amidst the labors of an exhausting practice, he found leisure to pursue his professional and scientific studies, and thus to keep pace with the advancing knowledge of his times. Few men ever exhibited a fresher interest or, possessed more extended and accurate information, upon these questions of philosophy and science which so deeply stir the age in which we live. Next to the topics of experimental religion, his mind reverted to them with the greatest freedom; and his searching criticism easily penetrated those fallacies by which the unwary are seduced into skepticism and unbelief. It was indeed refreshing to observe a mind naturally most highly endowed, and richly cultivated and disciplined, determining for itself the boundaries of human reason; in the province of faith bowing with the docility of a child before the oracles of God, yet moving with adventurous steps in the departments which lie open to the investigations of science and the deductions of logic.

During the progress of a religious revival in the year 1838, Dr. Picton was first brought to avow himself a Christian, and united with this Church under the Pastoral care of Rev. Joel Parker, D.D. How consistently, through twenty years, he has adorned the Gospel of our Lord Jesus Christ, it were useless for me in the presence of this Congregation, to proclaim. His professional calls, though often sudden and exacting, he was generally able so to arrange as not to interrupt his attendance upon the ordinances of the Sanctuary; and few men ever spoke from this desk without observing his fine intellectual face lighted up with thought and feeling, evincing the degree of his sympathy with the holy truths of the gospel. In 1845, he was chosen by the free and unanimous voice of this Church, to fill the responsible office of Ruling Elder, the duties of which he continued faithfully to discharge, during a period of thirteen years, till the day of his death. The maturity of his judgment, the clearness of his views, the strength of his convictions, the fevor of his piety, and his practical knowledge of mankind, pre-eminently fitted him for this high station; and those associated with him in office will cheerfully testify to the value of his counsels and to the prudence and zeal with which he labored to preserve the purity, and promote the piety, of the entire Church.

It was, however, in the seclusion of domestic life that Dr. Picton's character shone with peculiar beauty; as he was seen stripped of that reserve, which to some extent veiled him before the world. It was there, that relaxing the rigor of authority as his children advanced to maturity, he gently suggested the cautions of experience to temper the anticipations of hope. It was there he bore his household, morning and evening, before the Mercy-Seat above, and prayed as only a parent can plead for the eternal welfare of his children. But I must not rudely lift the curtain drawn around the sanctuary of his home; nor stir anew the grief of those who mourn over his loss with a bitterness of sorrow known only to themselves. Alas! we know how much death can do! At a single blow the tender husband, the loving father, the enterprising citizen, the genial friend, the prudent counsellor, the kind physician, the experienced instructor—all have fallen; all these relations laid prostrate at once in the grave which holds the manly form of one whom we shall no more see on earth. Yet at his grave we realize the unity of the Church of God, and that what is lost on earth, is treasured forever in Heaven. He who once mingled with us in the praises of this Sanctuary, is only transferred to a higher service, and engages in a more blessed worship among the Sons of God in the Temple above.

"Thou'rt gone to the grave, but we will not deplore thee;
Though sorrows and darkness encompass the tomb,
The Saviour has passed through its portals before thee,
And the lamp of His love was thy guide through the gloom.
Thou'rt gone to the grave, and its mansions forsaking,
Perhaps thy tried spirit in doubt lingered long;
But the sunshine of Heaven beamed bright on thy waking,
And the song that thou heard'st was the Seraphim's song."

It would be a pleasing task to fill up this meagre sketch, and to draw out at length the character and life of our deceased brother and friend. But the solemnities of this occasion demand of me a higher service than to pour the voice of praise into "the dull, cold ear of death." Had we power to bring him back to earth, he would be the first to put the crown from his own head upon that of his Master, and to say in the words of the text, "Brethren, be followers together of me, and mark them which walk so, as ye have us for an ensample." Especially might he address thus the class of which he was an honored representative, and persuade those of his own profession to take the yoke of Christ, and become obedient to the Gospel. I can pronounce no higher eulogy than to present him to your thoughts as the model of a pious Physician; and as his voice, to urge the claims of experimental religion upon the medical profession. It will be my aim therefore, in this discourse, to show the need of true piety as furnishing the brightest trait of excellence in the character of the Physician.

1. *The first consideration may be drawn from the principle of benevolence, in which the Science of Medicine undoubtedly originates.* It is impossible to determine what would have been the precise condition of the human race, had sin never been permitted to enter the world. Probably some of our most important and engrossing pursuits would be vacated, by the withdrawal of evils which now create the necessity for their continuance. We can at least confidently affirm that sin is the parent of those physical sufferings, which it is the object of Medical science to alleviate. This is the testimony of the Apostle Paul, "By one man sin entered into the world, and death by sin, and so death passed upon all men, for that all have sinned." In this passage, death is unquestionably a generic term, including all the sickness, and pain, and disease which find their consummation in death. No adequate explanation of them can be given, but they are judicial visitations upon man for his violation of the Divine law; so that we "have the sentence of death in ourselves, that we should not trust in ourselves, but in God which raiseth the dead." But these sufferings alone could never have originated the science of Medicine, unless they had been contemplated with an eye of benevolent compassion. The various diseases which ravage the earth, might have swept on in their destructive march, without an effort or even a wish to impede their course, but for the instinct of human sympathy which leads us to mourn over the sorrows of others. While, however, the holiness of God and the sanctity of his law are avenged in this punishment of sin through disease and death, his boundless compassion has furnished the remedies which human wisdom shall both discover and apply. To the minerals which are embedded in the earth's bosom, to the plants which grow in such magnificent profusion upon her soil, to the medicinal springs which bubble upon her hill-sides, he has imparted healing virtues, designed to soften the asperity of his judicial inflictions. It matters not by what happy accident the curative proprieties of any single fountain or plant were first discovered, for in it, as in a seedling, lay an undeveloped science. It was

"A spark struck into tinder to light the lamp of knowledge;
A slight suggestive nod to guide the watching mind;
A half seen hand upon the wall, pointing to the balance of comparison."

The same benevolence which before wept with unavailing sympathy over the sorrows of mankind, now sought eagerly

"To learn upon a hint, to find upon a clue."

Nature has been searched through her whole domain, yielding up her secret and long hoarded treasures to the relentless interrogations of science. Her products have been subjected to the tortures of analysis, till their hidden and essential principles have been eliminated and held apart. The human system has been laid bare to the inspection of the anatomist. until the seat of every organ has been defined, their respective functions accurately described, the reciprocal influence of each upon the rest carefully noted, and the action of every remedial agent experimentally demonstrated. In short, a benevolent curiosity has so pushed its researches, that in the lapse of ages, Medical science has grown into a grandeur of proportions which compels the homage of mankind. Whatever gibes it may encounter from the thoughtless, because at first empirical and uncertain in its researches, it never fails to vindicate itself in our hours of peril and of pain, and to wring the homage which it is the shame of many so reluctantly to pay.

These hints are scarcely superfluous if they recall your attention to the fact that however the profession of Medicine may take its spring in the sufferings of mankind, it owes its birth not less to that instinctive benevolence of the human heart, which cannot look upon distress without a wish to heal; and which walks up and down the earth, crying aloud, "is there no balm in Gilead? is there no physician there?"—Of this benevolence the Medical profession is the special exponent; beyond all others it represents, as a class, the power of this sentiment, than which there is none more noble in human nature. Other men *may* be benevolent—the physician *must* be. Other men do good upon *occasions*—the physician *habitually*, and by the very import of his calling. Other men relieve the destitute and the suffering by indirection, giving their substance for the erection of Hospitals and Asylums, distributing their alms by proxy; the physician, in his own person, gives the sweat of his brow and the labor of his hand, the anxieties of his soul and the toil of his intellect. Amid the sultry heats of summer as well as in the frost and sleet of winter—in the glare of noon, and in the midnight hour, when deep sleep falleth upon men—when worn with watching, and exhausted with fatigue—with the body struggling against incipient disease, and with the heart often bowing under the pressure of private sorrow; at all times the calls of humanity must be promptly obeyed; and they must go with equal cheerfulness into the hut of the pauper as into the palace of the prince. I am uttering, not one of the common-places of flattery, but the profound conviction of my own heart, when I say that no class in society renders so large an amount of unrequited service to their fellow men, as the Medical class. Their ethical code, which only gives formal expression to the purest impulses of our common nature, forbids the question of fee or reward to be raised, when first summoned to the bedside of the sick. Too often it is their misfortune to experience only the basest ingratitude of those, who in the first moments of relief would have kissed their hand as the kindest of deliverers. I know not who are more competent to bear testimony to the benevolent spirit in which the practice of Medicine is conducted, than the clergy. We go hand in hand with the physician into the chambers of suffering, and meet daily at the same bed-sides; and none should be able to judge better than we of the charity and kindness of Medical men. It is indicated in the noiseless tread with which they enter the darkened room; in the subdued and sympathising tones with which the sufferer is interrogated; in the gentle pressure of the hand upon the body as it is quivering with pain: in the cloud of anxiety which gathers upon the brow, as the disease works on defying their skill, to its fatal result; and in the overwhelming sadness with which as mourners they come to look upon those whom they vainly strove to save. Knowing how justly this tribute is deserved, I gladly seize this occasion to say, that through an official intercourse with Medical men, extending over nearly eighteen years, I have never found one of their number heartless and insensible—not one, however professionally cold and hard his exterior, who did not at proper times betray a true sympathy with the sufferings of his fellow men—not one who failed to represent the spirit of benevolence which underlies his calling—not one who did not deserve to be loved as a brother, and trusted as a friend. Though doubtless, like every class, made up of mingled good and bad, I am sure that in no profession are the practitioners more pervaded with the characteristic spirit of their calling than in this, whose very commission is to go about doing good.

But how much more beautiful this benevolence, if instead of being a blind instinct of our nature, it were sanctified by religion and wrought with in the soul as a grace of the Spirit? How much more would the kind physician realize the ideal of his own profession, if, ascending the stream of this benevolence to it source, he could first love his Maker with supreme affection, and then love his fellow men as the children of a common Father? How much more complete his sympathy, if, looking upon sorrow as the effect of sin, he could compassionate men in this the spring of all their miseries? How much more Godlike his vocation, if recognizing this brotherhood of race, he could come to them as the messenger of God—an angel of mercy, conveying that compassion which has its seat in the bosom of the Deity, and which through his agency is so freely distributed ? Following the example of Christ, whose life was a life of self-denial, and whose miracles were miracles of mercy, he would not be a solitary worker amid a field of thorns, but a co-worker with God in carrying forward his purposes of love to the lost sons of men. His charity would not then be a single and earth-born elegance, but the grateful utterance of his indebtedness to infinite grace ; and his professional career would be a sublime hymn of praise to One whose name is Love.

2. *True piety should adorn the Physician, as being the highest improvement of our spiritual nature, the recognition of which both the science and practice of Medicine equally compel.* It has been urged as a reproach against Medical studies, that they nourish a tendency to materialism. Dealing with the body as the seat of all diseases, tracing the immediate and material causes which produce them, and analyzing the properties of every remedial agent which can be expressed in the formulas of Chemistry, it is alleged that man comes to be viewed at last as only a higher species of animal, with a more delicate organization, it may be, but still corporeal and fleshly only, like all the beasts of the earth. It should not surprise us if superficial minds should rest in shallow conclusions like this. They are easily reached by a first induction, and do not require the large comparisons by which all final truths are attained, and which can only be instituted by broader intellects. But that the master minds in this noble profession, whose original research is continually widening the circle of Medical knowledge—that these should acquiesce in generalizations of this sort, is utterly incredible ; nor do I believe that the verdict of the Medical Faculty throughout the world would pronounce for a philosophic and scientific heresy so appalling as this. On the contrary, it falls within the observation of every intelligent physician, that man's soul rules the body in which it dwells, whose influence must be estimated in the the pathology of almost every form of disease. Often the body is overwhelmed by a complication of disorders, any one of which is competent to hurry it to the tomb ; yet the sinking frame is undergirded by an invisible and spiritual power, and the patient lives on apparently by the simple energy of his own will. Again, by an irrepressible sympathy with the mind, the body, almost free from disease, droops under the morbid influence which this spiritual tenant has the power to exert. Its juices are dried up from within, and after a feeble resistance it succumbs beneath the spiritual spell by which it has been withered away. It is precisely here, in the plane of this intersection of matter with mind, and in their reciprocal influence upon each other, that the most insoluble problems in Medical science are to be found. Dark as may be the mysteries which hang around the origin of many diseases, they are not hopelessly beyond the reach of scientific research. Future experiments may reveal the tests by which they shall be laid bare to Medical inspection. But who may hope to detect and imprison for scientific manipulation, that immaterial essence which we call the soul, or to lift the veil which conceals its inner working from the gaze of profane and curious eyes? It is only by collecting and classifying the facts of our own consciousness, and by eliminating thus the laws of our spiritual economy, that the secret processes of thought and feeling can be traced. These metaphysical inquiries are so far from being impertinent, however they may be derided by some, that the skillful practitioner is often guided by them alone in determining the character of many affections of the body, which otherwise were wrapt in impenetrable obscurity. I am greatly in error, if this is not one of the special advantages enjoyed by the family physician over the occasional Medical adviser. The former has opportunities of which he dilligently avails himself, to study, not only the idiosyncracies of the physical constitution, and the traditional morbid tendencies which descend from parent to child, but also those intellectual and moral characteristics which continually interpose to modify, in the individual, the operations of disease. It is for this purpose that he visits his patients in seasons of health as well as of sickness; whilst apparently solacing himself with the delights of social intercourse and friendship, he is in fact drawing out all the features of his patient's character in its sound and normal condition, and is often able to predict with almost unerring certainty how, under the invasions of disease, each bodily organ shall be affected.

The Physician cannot, therefore, ignore man's higher and spiritual nature without exhibiting himself to the world a mere empiric. He cannot rise into loftier walks, nor solve the deeper problems, of his own profession, after discarding principles that are fundamental in his science. As a mere practitioner he must often fail of success when called to "minister to a mind diseased." It is difficult to restrain the expression of disgust which is excited by so low a view of human nature. As the philosophic materialist, in his repudiation of the human soul, spunges out that image of God in which man was made, and finds himself shunned as an apostate from mankind; so the Medical materialist, by lowering an august profession to the level of simple quackery, should not be surprised to find himself dismissed to the grade of the petty hucksterers who vend their nostrums in his own art. But that materialism forms no part of the medical creed is abundantly shown from the ready access which clergymen gain to the bedside of the sick and dying. With very rare exceptions, Ministers of the Gospel who possess ordinary discretion, are hailed as the Physician's most useful allies. Obvious reasons must exist in the nature of the disease, or want of confidence must be felt in the Pastor's prudence, in order to justify his exclusion from the chamber of sickness What is this but a free confession that man's immortal part rules the mortal; or that his immortal interests should not be perilled through neglect?

This recognition by Physicians of man's spiritual and religious nature sweeps on to grave and practical conclusions How shall the Physician compass the highest excellence in his chosen calling, if he cannot appreciate the cravings of the human soul, nor understand the blessings by which those cravings may be met? Simply therefore to reach the highest type of professional skill, he must thoroughly comprehend the whole complex nature of man; and know by experience the value of religion in meeting the wants of the human soul. How shall he, if ungodly, bring himself in closest sympathy with that large and increasing class of patients who feel the transcendent importance of those very interests which he overlooks or denies? Let it be remembered that this Gospel of Jesus Christ, in its sublime march to universal conquest, is gathering larger multitudes beneath its sway; and whatever respect may be shown to simple professional skill, true piety with the pious, will always be a bond of sympathy. If too, this soul is of paramount value to the patient, is it of less moment to the Medical adviser? If in all his professional practice he defers to it as the power which should rule over the corporeal part, why should not its supremacy be acknowledged in himself? He is not less than others a full man—and that religious nature which commands his homage in others, claims as much when seen in himself. Each patient becomes thus a monitor, bidding him find the highest improvement of his own character in that true piety, which is but the lost image of God restored to the soul. It is plain, therefore, that Christianity presses its claims upon the Medical profession by their own confession of judgment; and that the cap-stone of excellence is not added, and the climax of perfection reached, until the fear of the Lord crowns their other attainments, and consecrates the whole to the glory of God.

3. *The Physician in the practice of his art, is continually led up into the presence of God, which should of itself impart to his character a religious tone.* The realities of the eternal world perpetually cast their shadows upon his path; and making due allowance for the effect of familiarity in blunting the sensibilities, it is hard to see how he can wholly escape from the influence of the solemn scenes in which he is always an actor. He is compelled daily to witness the impotency of this world in securing the happiness and well-being of its votaries; how a single spasm of pain withers every enjoyment, and how complete a mockery all the advantages of wealth and position appear in the chamber of death. He cannot but see how feeble is the tenure by which the gifts of earthly fortune are held; and with what reluctance the hand uncloses to release the bounties upon which the grasp had been growing tighter in the season of prosperity. He hears the melancholy confessions which in that honest hour are extorted from hearts long estranged from God, and listens to the earnest testimony which is then borne to the transcendent importance of religion. That spell of fascination by which the world holds its victims, while flaunting its gay visions before the eye, is effectually broken as the curtain is partially drawn aside which conceals the retributions of Eternity.—If too, on the one hand, the vanity of this world is so painfully demonstrated, on the other, the reality and power of true religion are equally illustrated. It is often his privilege to hear the language of exulting hope from the lips of those who by nature are the most fearful and timid of our race; and he cannot but feel that the notes of triumph which die away in the last panting breath of the Christian swell out immediately in the music of that eternal Song which is heard forever in Heaven. Thus, to appropriate a striking metaphor of John Foster's in one of his inimitable letters, the Physician, mingling in those scenes of death, stands "at the high altar of Eternity to pronounce the inviolable oath;" he engages in "a kind of sacrament inconceivably solemn, at which he is summoned by the voice of Heaven to pledge himself in vows of irreversible decision." Alas! that these pregnant instructions should fall upon ears which heed them not! that he should turn away his feet from those hallowed shrines in the vestibule of Eternity, to chase, eagerly as before, the fleeting phantoms of earth! that he should treasure only with a romantic interest these reminiscences, which in fact are foreshadowings of the unknown future which he must so soon explore.

But there is another way by which the practitioner of Medicine is conducted before God: he is surrounded by mysteries in the prosecution of his art, which are always suggestive of the Deity. It is the fashion of many to cry out against mystery in religion, as though it was peculiar to that revelation of God which is made in the Sacred Scriptures. Intoxicated with the partial knowledge he has acquired, man grows insolent in his demands, desiring to be himself as God, knowing both good and evil. Yet he can enter upon no path of inquiry without finding all the resources of his reason baffled. "It is the glory of God to conceal a thing;" and the mysteries both of nature and of grace are but the veil which the Deity throws around Himself, fill us with awe as we stand in His august presence, and to raise the anticipations of fuller disclosures in a better world. Perhaps, in no one of the learned professions are we more confronted with the supernatural, than in the Medical. Its literature is occupied with discussions revolving for ages around the same centres, whose heart has never yet been penetrated. What life is, which it is the aim of this science to conserve—where in the human body it holds its seat—how it should pervade with equal energy every portion of our frame—why it should depend upon the union of matter with spirit, and disappear instantly when this connexion is severed—by what ties the soul is held in companionship with that body which it informs and actuates–what is disease, and how it should be originated–what are the laws of its propagation, how transmitted from sire to son through succeeding generations—how it should undergo such modifications, as almost to lose its essential character, and enter into new combinations, baffling even the experience and skill of the most learned; all these questions need only be propounded to show the haze of mystery enveloping this noble art. However confident the man of science may be that all these matters are determined by certain fixed laws, yet the subtlety of these laws, escaping hitherto all research, requires the intervention of God by whom they are enacted and upheld. Should future scrutiny penetrate their working, it is only to resolve them into larger and more comprehensive rules; enthroning the Deity in adoring worship.

As in the theory, so again in the practice of Meicine, we come continually in contact with God. To-day, a case presents itself in which from all that appears to view, the Physician would antecedently say, the man ought to recover; yet in spite of these hopeful prognostications and in defiance of all the skill and science which can be brought to bear, the patient dies. Tomorrow, another case occurs in which from all the symptoms no other judgment can be formed but that the man must die; yet in the face of this prediction, and in opposition to all the aggravations of the disease, the crisis is happily passed and the patient survives. What thoughtful practitioner can fail to recognize in these daily recurring facts the existence of another Power which is at last the arbiter of life and death; and that upon the blessing of this invisible but superior being, his own success in the healing art depends? In whom then more than in the Physician should the devotional sentiment be elicited? and how naturally does true piety seem to crown the character of one, who, in every step of his career finds himself touching his Maker?

4. *The final argument urging the claims of religon upon Medical men, is drawn from the opportunities they enjoy of doing good to the souls of men.* Physicians more freely than other men move through all the grades of Society, mingling with high and low, the virtuous and the vile. In other pursuits men are more or less restricted to that circle in which Providence has cast their lot; or if they cross the artificial zones of society, it with a casual and distant intercourse. In this particular, Clergymen make the nearest approach to Physicians, in that they both occupy at once all the points in the social scale and are equally at home with all ranks. Yet the latter have preminently the advantage in gaining access to multitudes, which however coveted, is often denied to the former. The vicious habits of many remove them from clerical influence by an interval over which it is difficult to pass. When broken down under their excesses, the man in black is precisely the last person whom they wish to see. His presence alone conveys a rebuke whose sting is felt as it awakens all the pangs of remorse; and his approach is too often considered as the death warrent which hastens the day of doom. None of these associations embarrass the Physician. His entrance into the chamber of sickness is welcomed as that of a friend and deliverer; and his mission is not regarded as one of rebuke, or even of admonition. But whilst soothing the pains of the sufferer, what a golden opportunity is offered of pointing the weary soul to the rest that is in God, and directing the penitent to that Saviour who offers pardon through His own blood. But these opportunities of usefulness draw along consequences that are momentous as the bar of judgent. Little as men may reck of it, they will be held to the strictest account for all the good they omitted to do; and no division of labor by which the duties of life are parcelled out can absolve any from the obligation of doing good to all men as they have opportunity. From the nature of his calling, the Physician is always touching wires that vibrate in Eternity; and woe be to him, who reckless of spiritual things permits a souls to slip through his hands without a warning of the destiny to which it is hastening. I need not pause to show how all this infers the need of true piety in the Physician, who would meet faithfully all the trusts involved in his profession. To win a soul from death requires far more than the cold testimony of one who speaks only by report. It needs the heart of love and the tongue of fire in one who has himself "felt the powers of the world to come," to make a true evangelist to the soul struggling at the last moment with its everlasting fate; for which reason the Physician, who is often the only messenger of God to such a soul, should be a warm and zealous Christian.

In venturing these suggestions to gentlemen of the Medical profession, I trust I shall not be charged with presumption. It is seldom they are addressed as a class; and in a Pastor's ordinary ministrations it might appear alike obtrusive and invidious to single them out as objects either of criticism or counsel. But on this occasion when we meet to pay a just tribute of respect to the memory of one whom we equally honor, who was as conspicuous for his Christian virtues as for his professional attainments, it seemed proper to hold him forth as the type and model of his class. He was a beautiful exponent of all that I have attempted this day to discuss. What can I say more in his praise than that you "mark them which walk so as you have him for an ensample;" especially in this, that you equally exemplify what it is to be a *Pious Physician.* It is little for me to say that I profoundly honor your noble profession—a profession venerable for its great antiquity, ennobled by the genius which in every age has enriched its treasures, and surpassingly beautiful in the principles of charity and beneficence in which it is founded. Let it only be crowned with grace; let religion hallow it with its blessing; let true piety adorn those who pursue it; let its mission be one of salvation to the bodies and souls of men, and its rewards will be laid up to be enjoyed beyond the grave.

[*N. O. Med. News & Gaz.*

